# A Novel MVA Vectored Chikungunya Virus Vaccine Elicits Protective Immunity in Mice

**DOI:** 10.1371/journal.pntd.0002970

**Published:** 2014-07-24

**Authors:** James Weger-Lucarelli, Haiyan Chu, Matthew T. Aliota, Charalambos D. Partidos, Jorge E. Osorio

**Affiliations:** 1 Department of Pathobiological Sciences, School of Veterinary Medicine, University of Wisconsin-Madison, Madison, Wisconsin, United States of America; 2 Takeda, Inc., Madison, Wisconsin, United States of America; Centers for Disease Control and Prevention, United States of America

## Abstract

**Background:**

Chikungunya virus (CHIKV) is a re-emerging arbovirus associated with febrile illness often accompanied by rash and arthralgia that may persist for several years. Outbreaks are associated with high morbidity and create a public health challenge for countries affected. Recent outbreaks have occurred in both Europe and the Americas, suggesting CHIKV may continue to spread. Despite the sustained threat of the virus, there is no approved vaccine or antiviral therapy against CHIKV. Therefore, it is critical to develop a vaccine that is both well tolerated and highly protective.

**Methodology/Principal Findings:**

In this study, we describe the construction and characterization of a modified Vaccinia virus Ankara (MVA) virus expressing CHIKV E3 and E2 proteins (MVA-CHIK) that protected several mouse models from challenge with CHIKV. In particular, BALB/c mice were completely protected against viremia upon challenge with CHIKV after two doses of MVA-CHIK. Additionally, A129 mice (deficient in IFNα/β) were protected from viremia, footpad swelling, and mortality. While high anti-virus antibodies were elicited, low or undetectable levels of neutralizing antibodies were produced in both mouse models. However, passive transfer of MVA-CHIK immune serum to naïve mice did not protect against mortality, suggesting that antibodies may not be the main effectors of protection afforded by MVA-CHIK. Furthermore, depletion of CD4^+^, but not CD8^+^ T-cells from vaccinated mice resulted in 100% mortality, implicating the indispensable role of CD4^+^ T-cells in the protection afforded by MVA-CHIK.

**Conclusions/Significance:**

The results presented herein demonstrate the potential of MVA to effectively express CHIKV E3-E2 proteins and generate protective immune responses. Our findings challenge the assumption that only neutralizing antibodies are effective in providing protection against CHIKV, and provides a framework for the development of novel, more effective vaccine strategies to combat CHIKV.

## Author Summary

Chikungunya virus (CHIKV) has recently re-emerged from Africa to cause disease outbreaks in Asia, Europe, and more recently the Caribbean. The virus is transmitted by *Aedes* mosquitoes and causes a disease that is characterized by high fever and incapacitating joint pain that can cause great personal and economic loss. At present, no approved vaccine or antivirals are approved against CHIKV. In this study, we developed a novel CHIKV vaccine that is vectored by Modified Vaccinia virus Ankara (MVA), an attenuated vaccine vector which has been shown to be safe in humans and induce a strong immune response. The vaccine expresses the E3 and E2 proteins of CHIKV, the latter of which is thought to be the main mediator of protection. The vaccine was effective in two mouse models and protected against all markers of disease tested despite the absence of high levels of neutralizing antibodies, the gold standard of protection. Depletion of CD4+ T cells from vaccinated mice resulted in loss of protection, implicating these cells in the protection induced by the vaccine.

## Introduction

Chikungunya virus (CHIKV; *Togaviridae, Alphavirus*), the etiologic agent of Chikungunya fever is an emerging pathogen that has recently caused several severe outbreaks throughout Africa and Southeast Asia [1]–[3]. Large outbreaks have occurred on La Réunion island (an overseas department of France), Mauritius, Sri Lanka, and India, among others [4]. In addition, autochthonous transmission has been seen in Europe, with outbreaks occurring in both Italy and mainland France [5], suggesting temperate climates can support virus transmission. Furthermore, as of January 2014, the European Centre for Disease Control has confirmed 70 cases of CHIKV on the Caribbean islands of St. Martin, Martinique, Guadeloupe, and Saint Barthelemy, with many more suspected, indicating spread to continental America is possible. CHIKV is transmitted to humans by *Aedes aegypti* and *Aedes albopictus* mosquitoes, the latter of which has been an important vector in many of the recent outbreaks due to mutations in the envelope genes of the virus that allow for more efficient transmission [6]–[9]. CHIKV causes a dengue-like illness associated with fever, rash and joint pain and was first described in modern day Tanzania in 1952 [10]. The term Chikungunya is derived from the Makonde word meaning “that which bends up” and describes the posture of an infected individual [11]. Recently, the U.S. Army developed a live-attenuated vaccine candidate, called CHIK 181/clone 25 or 181/25, but it caused transient arthralgia in a small number of volunteers during phase II clinical trials [12], [13]. Experimental subunit [14], recombinant viruses [15]–[17], and VLP [18] based vaccines have also been described which are currently at various stages of preclinical or clinical development, however there currently are no licensed vaccines or antiviral treatments available for CHIKV.

An alternative approach for developing CHIKV vaccines is the use of viral vectors. A complex adenovirus expressing the complete CHIKV structural poly-protein has been described and was shown to be immunogenic and protective in mice [19]. However, safety concerns regarding adenovirus based-vectors may limit this approach [20]. In contrast, Modified Vaccinia virus Ankara (MVA) has been tested in over 120,000 humans and was proven to be highly safe and effective in protecting against smallpox [21], [22]. MVA was attenuated by over 500 passages of Vaccinia virus (VACV) in chicken embryo fibroblasts (CEFs), which resulted in large deletions of its genome that restricted its host-range [23], [24]. During passaging, MVA lost the ability to productively infect mammalian cells, leading to abortive replication [25]. VACV, which is far more reactogenic than MVA [26], [27], has been used previously against other alphaviruses, namely Sindbis and Venezuelan equine encephalitis (VEEV) viruses, providing robust immunity against the latter, suggesting poxviruses as suitable vectors against alphaviruses [28]–[33]. Despite the presence of high levels of neutralizing antibodies elicited by most of the VACV vectored VEEV vaccine candidates, they were ineffective in providing protection against airborne infection, suggesting they were unable to elicit a sufficient T cell mediated immune response, which has been shown to be critical for protection against lethal VEEV encephalitis [34], [35]. MVA, like its parent virus VACV, has been extensively tested as a vaccine vector, expressing viral, bacterial or parasite antigens and has been shown to induce both humoral and cell-mediated protective immune responses [25], [36]–[42]. Furthermore, MVA can be delivered effectively by different routes and has much greater stability than most other live virus based vaccine approaches [43]. Additionally, because MVA undergoes only abortive replication in mammalian cells, vector stability is not a problem as only one infection cycle occurs [25].Therefore, an MVA vectored CHIKV vaccine would be an attractive option for resource-limited countries, where the majority of CHIKV infections occur. Additionally, Volz and Sutter (2013) have targeted MVA as an ideal vector for safe next generation vaccines, with CHIKV being mentioned specifically as an attractive candidate pathogen [44]. Furthermore, Garcia-Arriaza et al. (2014) successfully used MVA for expression of the entire CHIKV structural protein as a vaccine candidate, indicating MVA can provide effective immunity against this virus [45].

In this report, we describe the construction and immunological evaluation of an MVA-based CHIKV candidate vaccine based on the E3-E2 proteins. The vaccine was tested in two mouse models- one immunocompetent and the other lacking α/β interferon signaling (A129)- and was uniformly protective. Mice deficient in α/β interferon signaling have been used for many viruses and have been shown repeatedly to be a good model for CHIKV, providing many similarities to human infection [46]–[48]. Complete protection against mortality was provided when MVA-CHIK was administered in a prime-boost regimen. In addition, it provided 80% protection against mortality in this highly immunocompromised mouse model after only 11 days post-vaccination. Surprisingly, while playing a role, neutralizing antibodies did not appear to be necessary for protection against CHIKV, contrary to other recent reports [49], [50]. Most importantly, depletion of CD4^+^ T cells in vaccinated mice resulted in loss of protection, with 100% succumbing to infection upon challenge with wild-type CHIKV, indicating an indispensable role of MVA-CHIK immune CD4+ T cells in protection.

## Materials and Methods

### Ethics statement

This study was carried out in strict accordance with the recommendations in the Guide for the Care and Use of Laboratory Animals of the National Institutes of Health. The IACUC protocol (Protocol #V01380) was approved by the Institutional Animal Care and Use Committees of the University of Wisconsin.

### Cell culture and viruses

African Green Monkey kidney cells (Vero, ATCC #CCL-81) and baby hamster kidney cells (BHK-21, ATCC # CCL-10) cells were maintained in Dulbecco's modified Eagle medium (DMEM; Gibco, Carlsbad, CA) supplemented with 5% fetal bovine serum (FBS), 100 U/ml of penicillin, 100 µg/ml of streptomycin, and 2.5 µg/ml amphotericin B, and incubated at 37°C in 5% CO_2_. Chicken embryo fibroblasts (CEFs) were obtained from Charles River (Charles River Laboratories International, Inc., Wilmington, MA) and maintained in OptiMEM (Invitrogen, Carlsbad, CA) supplemented with 10% FBS under the same conditions. Chikungunya virus strain La Reunion (CHIKV-LR; GenBank: DQ443544.2) was used for both the construction of recombinant poxviruses and challenge experiments. The virus was kindly provided by Dr. Scott Weaver (University of Texas Medical Branch, Galveston, Texas). The CHIK-IRES vaccine candidate has been previously described [51] and was used in this study as a positive control. Briefly, the vaccine was constructed by replacing the subgenomic promoter in a cDNA CHIKV clone with an internal ribosome entry site from encephalomyocarditis virus (EMCV-IRES) and has shown to be effective and safe in mice [51] and non-human primates [52]. The MVA virus used in these studies was obtained through BEI Resources (NIAID, NIH,ref # NR-727). A recombinant MVA virus expressing GFP (herein called MVA-GFP) was based on the wild-type MVA and its construction has been previously described [37], [53].

### Construction of recombinant MVA vaccines

**Figure 1:**
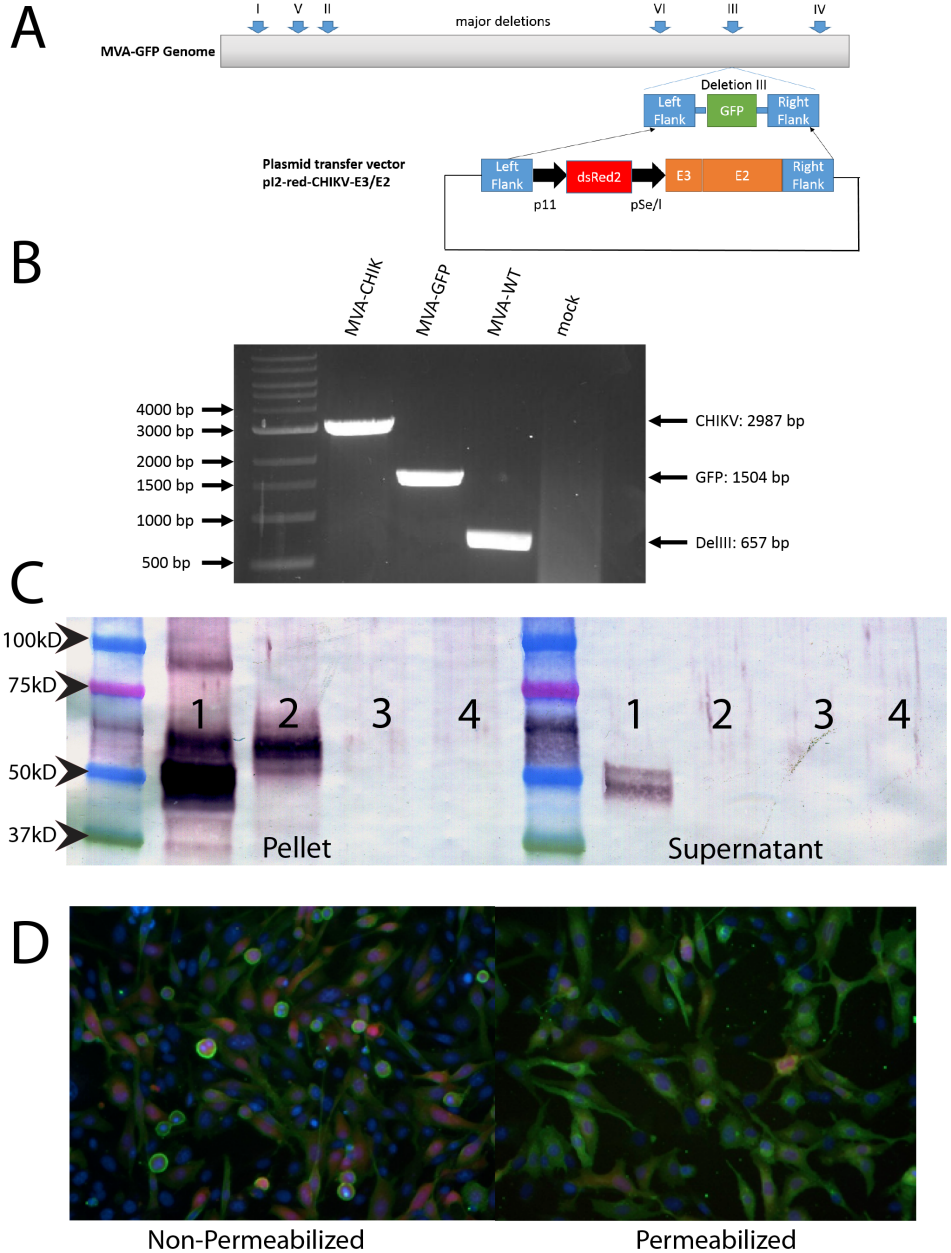
Generation and characterization of candidate MVA-CHIK vaccine virus. Diagram of the MVA-GFP genome and transfer vector used for homologous recombination for creation of MVA-CHIK. The different areas where deletion occurred in the vaccinia virus genome to create MVA are shown. MVA-CHIK was created by transfecting the plasmid containing CHIKV p62 under control of a se/l promoter which also expressed dsRed2 under control of a p11 promoter. This plasmid contains flanking regions to deletion III (DelIII) which is where the recombinant gene was inserted into the genome with a fluorescent marker (A). PCR analysis of the DelIII region. Viral DNA was extracted from purified, final virus stocks of MVA-CHIK, MVA-GFP and MVA-WT. PCR was performed using primers specific for the DelIII flanking regions. A DNA ladder is included for comparison of size (B). Monolayers of CEF cells were infected with recombinant MVA-CHIK viruses at MOI of 5 or 10 PFU/cell for Western Blot or Immunostaining, respectively. After 24 h post infection, cells were harvested and subjected to SDS-PAGE followed by western blot analysis (C) or fixed with 2% PFA as described in the methods. Both the cellular pellet and supernatant were analyzed for expression of CHIKV E3/E2. The order is the same for both and is as follows, wild-type CHIKV (1), MVA-CHIK (2), MVA-GFP (3), and mock infected cells (4). Following fixation, cells for immunostaining were either allowed to remain intact or permeabilized with triton X-100 (D). Anti-CHIKV polyclonal rabbit was used for primary staining for both assays. Green represents CHIKV E2 positive cells, red is dsRed protein produced by the virus and blue is nuclear staining with Hoechst.

For the production of recombinant MVA vaccines, the CHIKV E2 and E3 genes (hereafter called p62) were PCR amplified using Phusion High-Fidelity DNA polymerase (New England Biolabs, Ipswich, MA) and cloned into a specially modified poxvirus transfer vector, pI2-Red [37]. E2 was chosen because it has been previously demonstrated that most neutralizing antibodies mapped to epitopes in this region [49], [54], [55]. E3 has not been strongly associated with protection; however, it was included to allow proper folding of the E2 protein [56]. The expression of p62 was controlled by a synthetic early/late Vaccinia virus promoter and used the Vaccinia virus transcription terminator, which have been previously described [57]. The vector used in this study contained flanking sequences that when transfected into MVA-GFP infected cells can recombine into the Deletion III region of the viral genome [58]. This vector contains DsRed under the control of a late p11 promoter to allow for visual based selection and permits an easy distinction between recombinant (red) and wild-type (green) viruses (Fig. 1A).

Recombinant MVA-CHIK viruses were generated as described previously [59], [60]. Briefly, CEFs were seeded into six-well plates the day before transfection and then infected at a multiplicity of infection (MOI) of 0.05 PFU/cell for 1.5–2 hours with MVA-GFP. Cells were then washed with 1× PBS and transfected with appropriate transfer vectors using FuGENE HD (Roche Diagnostics, Indianapolis, IN) following manufacturer's protocol. Cells were monitored for the presence of red fluorescence 24 hours after transfection. At 48–72 h post-transfection, monolayers were harvested, centrifuged at 500×g for 5 min at 4°C and cells disrupted by freeze–thaw (3 times) followed by sonication (2 times for 15 s using a cup sonicator). The disrupted cell extracts containing recombinant viruses expressing DsRed were plated onto fresh CEF cells and overlaid with 0.8% agarose. After 48–72 h, recombinant virus-generated plaques were detected by observing fluorescence and picked into 300 µL media with a sterile filter pipette tip. The cell/virus samples were subjected to freeze thaw and sonication (as described above) and plated to continue passaging. Plaques were passaged until no wild-type (GFP expressing) virus was observed at which point PCR was performed to confirm the presence of only recombinant virus [61]. High titer virus stocks were prepared from PCR positive cultures and recombinant MVA-CHIK viruses were further characterized. PCR analysis was performed on final virus stocks to ensure genetic homogeneity and stability. DNA was extracted using the quick-gDNA Miniprep kit from Zymo Research per manufacturer's instructions (Zymo Research, Irvine, CA). PCR was then performed using Phusion polymerase with deletion III specific primers (Forward 5′-atgcggcacctctcttaa-3′, Reverse 5′-tgggctccttataccaagca- 3′).

### *In vitro* characterization of recombinant MVA-CHIK vaccines

Western blot analyses were used to determine the *in vitro* expression patterns of MVA-CHIK constructs. For this purpose, BHK-21 cells were seeded at 3.0×10^5^ cells/well into six-well plates and 24 hours (hr) later infected at an MOI of 5 PFU/cell in OptiMEM in the absence of FBS. At 24 hr after infection, cells were harvested and lysed using Radio-Immunoprecipitation Assay buffer (RIPA; 150 mM NaCl, 1.0% NP-40, 0.5% sodium deoxycholate, 0.1% SDS, and 50 mM Tris, pH 8.0) at 4°C for 30 minutes (min) under gentle agitation. Lysates were then centrifuged for 20 min at 12,000 rpm and supernatant was collected for further analysis. Supernatants were diluted into 2× Laemmli buffer (Bio-Rad, Richmond, CA), heated at 95°C for 5 min and 50 µL were loaded into a Bio-Rad 4–20% precast gel. Gels were transferred to a nitrocellulose membrane using Trans-Blot Turbo Blotting System following manufacturer's instructions. Polyclonal serum obtained from specific-pathogen-free (SPF) rabbits inoculated with a vaccine strain of CHIKV was used to probe blots at a dilution of 1∶5000 and developed using BCIP/NBT alkaline phosphatase system (Bio-Rad).

BHK-21 cells were used for immunocytochemistry (ICC) experiments. One day prior to infection, 2×10^5^ cells were plated onto glass coverslips, which had been placed inside of 24 well tissue culture plates. The following day, the cells were infected at an MOI of 10 PFU/cell. Infection was allowed to progress for 24 hrs and then fixed using 2% paraformaldehyde (PFA) solution in phosphate buffer. For permeabilization, cells were treated with 0.5% Triton X-100 for 10 min at 4°C, followed by 50 mM NH_4_Cl in PBS for 10 min at room temperature (RT), and then blocked with PBS containing 10% bovine serum albumin (BSA) for 3 to 4 hr at RT. Cells were then incubated overnight at 4°C with a 1∶1000 dilution of anti-CHIKV polyclonal rabbit serum. Cells were subsequently washed three times with PBS containing 0.05% Tween and then incubated with a 1∶2000 dilution of anti-rabbit IgG Alexafluor-488 for 45 min at RT. Cells were mounted using Vectashield mounting medium (Vector Laboratories). Images were acquired using an Evos microscope with attached camera.

ELISAs were performed as described by Brewoo *et al.*, 2010 [37]. Briefly, 96-well ELISA plates were coated with purified CHIKV (0.5 µg in 100 µL carbonate buffer, pH 9.6 per well) at 4°C overnight. Coated plates were washed twice with 0.05% Tween-20 in PBS (washing buffer) and incubated with blocking buffer (1% BSA in PBS) at RT for 1 hr. Serum samples then were serially diluted from 1∶100–1∶12,800 in ELISA diluent (0.1% BSA in washing buffer) and added in triplicate to the prepared ELISA plates and plates were incubated at RT for 1 hr. Known negative (uninoculated) and positive serum samples from mice inoculated with CHIK-IRES from previous studies were used as controls. After washing, 100 µL per well of a 1∶10,000 dilution of horseradish peroxidase (HRP)-conjugated rabbit anti-mouse IgM+G (Abcam Inc, Cambridge, MA) was added to each well and incubated at RT for 1 hr. Plates were washed, and 100 µL per well of tetra-methyl-benzidine (TMB) chromogen (Sigma, St Louis, MO) was added to each well and incubated in the dark for 5 min. The reaction was then stopped by adding 100 µL per well of 2 mM H_2_S0_4_. Colorimetry was measured using an ELx800 absorbance microplate reader (BioTek, Winooski, VT) at test wavelength of 450 nm and a reference wavelength of 630 nm. The highest dilution that was positive (exceeded the mean of known negative serum samples plus three standard deviations) was considered the endpoint, and its reciprocal value was recorded as the titer.

### Animal experiments

Groups of four- to six-week-old female BALB/c mice (Harlan Sprague Dawley, Indianapolis, IN) or six- to ten-week-old mixed gender α/β interferon signaling deficient mice (A129) received either primary only or primary and booster immunizations (28 days apart) with each vaccine candidate via intradermal (i.d.) injection into the hind, left footpad. A dose of 1×10^7^ TCID_50_ units in 50 µL was used for all MVA vaccinations. The dose chosen is either lower than or consistent with many previous reports of MVA vectored vaccines [62]–[64]. Negative control groups were immunized with MVA-GFP at the same dose. As a positive control, mice were vaccinated with experimental control vaccine virus CHIK-IRES [17] at a dose of 10^4^ TCID_50_ units. At either 11 days post-prime or two weeks post-boost (where applicable), all animals were challenged with wild-type CHIKV-LR by i.d. inoculation of 10^4^ (BALB/c) or 10^2^ (A129) TCID_50_ units in 50 µL into the hind, left footpad. Mice were bled prior to boost and prior to challenge following vaccination to monitor levels of neutralizing and anti-virus antibodies. Passive transfer studies were performed using A129 mice as previously described [37]. Briefly, serum was collected from vaccinated BALB/c or A129 mice and equal volumes from each sample were used to create a pool of sera for each mouse strain separately. 100 µL (BALB/c) or 200 µL (A129) of this inoculum were then injected into naive mice intraperitoneally (i.p.). Serum from CHIK-IRES vaccinated A129 mice (100 µL) was used as a positive control. 24 hours after passive transfer, mice were challenged in the same manner as described above. Following challenge, mice were bled three days consecutively to monitor viremia via the maxillary vein. With the exception of the BALB/c experiment, all animal experiments were repeated at least once, with similar results.

### *In vivo* depletion of T-cells

For depletion studies, groups of six- to ten-week-old A129 mice (n = 5), were vaccinated i.d. with 10^7^ TCID_50_ units of MVA-CHIK on day 0 and boosted day 28. On days 39 and 41 post priming, immune mice were treated i.p. with 100 µg of anti-CD4 mAb (GK1.5) or 250 µg of anti-CD8a mAb (2.43) (Bio X Cell). Control groups included untreated immune and non-immune animals. Each treatment group was challenged i.d. with 500 TCID_50_ units of CHIKV-LR three days later. Mice were further treated with depleting antibodies 3, 7 and 10 days post-challenge. The depletion efficiency in PBMCs on the day of infection was more than 99% for both T cell subsets as assessed by flow cytometry by staining with anti-mouse CD4 FITC (RM4-5), anti- mouse CD8a PerCP (53–6.7) mAbs (from BD Bioscience) (data not shown). Mice were monitored for morbidity and mortality for two weeks.

### Histopathology

Histopathology was performed on tissues collected from A129 mice 7 days post infection (d.p.i.). Footpad and leg tissues were severed from euthanized mice, cut in half to expose the tissues, and fixed in 4% buffered PFA for two days. Hind limbs were then decalcified in 4% PFA containing 14% EDTA for 3–4 weeks, changing decalcification solution weekly. Tissues were paraffin embedded, sectioned and stained with hematoxylin and eosin (H&E). Pictures were taken using a Sony NEX-5N camera attached to a Nikon microscope.

### Virus quantification and serology

Blood samples collected from mice following challenge were used to assess viremia. Viremia was assessed via end point dilutions in 96 well plates seeded with Vero cells (seeded the night before at 2×10^4^ cells/well) and was expressed as tissue culture infectious dose 50 (TCID_50_) per ml. For prime only studies a serum sample was taken ten days post-vaccination to measure neutralizing antibodies. For studies with prime and boost serum samples were collected on days 28 and 42 post-primary vaccination. Neutralization titers were determined using a TCID_50_ based assay that was modified from Grosfeld *et al.* [65]. Briefly, sera were incubated at 56°C for 60 min to inactivate complement and then serially diluted two-fold starting at a dilution of 1 in 5. Dilutions were performed in DMEM as described above except containing only 2% FBS. Subsequently, 100 TCID_50_ units of CHIKV were added to each well in the same dilution media. The virus/serum mixture was then incubated at 37°C for 1 hr. Following incubation, 100 µL of the mixture was added to 96 well plates seeded with Vero cells and monitored for 3–4 days for presence of cytopathic effects (CPE).

### Flow cytometric analysis of T cell responses

T cell responses were analyzed as previously described [50], [53]. Briefly, A129 mice were euthanized 2 weeks post-boost. After red blood cell (RBC) lysis, single-splenocyte suspensions were resuspended in RPMI-1640 medium supplemented with 10% FBS 1× penicillin/streptomycin and 0.14 mM of β-mercaptoethanol. Splenocytes were stimulated with three CHIKV peptide pools that cover the entire protein sequences of E3, E2 and NSP2, respectively. The peptide pools were used at a concentration of 1 µg/well separately in 200 µL total volume for 16 h in the presence of Brefeldin A. Peptide pools were a generous gift from Dr. Daniel Streblow [66] and were synthesized by Thermo Fisher Scientific. Cells were stained intracellularly for IFN-γ APC (XMG1.2), IL-2 PE (JES6-5H4), TNF-α PE (MP6-XT22) and CD40L (MR1) after surface staining of CD4 FITC (RM4-5) or anti-mouse CD8a PerCP (53–6.7). The samples were acquired on a BD FACSCalibur and analyzed with FlowJo v7.6.5 (Tree Star).

### Statistical analysis

GraphPad Prism 6 software (La Jolla, CA) was used for all statistical analyses. Statistical analysis of viremia levels were performed using an unpaired T-test with Welch's correction for unequal variance. Survival analysis was performed to assess vaccine effectiveness against; reported *P*-values are from the Mantel-Cox test.

## Results

### Characterization of MVA-CHIK recombinant vaccine: PCR analysis, protein expression and cellular localization

Genetic homogeneity of final virus constructs was analyzed by PCR. Purified viral DNA was used as a template for amplification by primers specific for the flanking regions of deletion III. The presence of E3/E2 was confirmed in MVA-CHIK by a band at the expected size of roughly 3 kb (Fig. 1b) which indicated the maintenance of the insertion throughout plaque selection. MVA-GFP (∼1500 bp) and wild-type MVA (657 bp) were included as controls. In addition, DNA sequencing confirmed that no genetic alterations were present in the final construct (data not shown).

The expression of p62 antigen by the MVA-CHIK recombinant viruses and wild-type CHIKV was monitored by immunoblot analysis. Cell pellet lysates and supernatants from infected CEFs were tested for protein expression (Fig. 1c). The p62 (62 kD) protein was detected at 24 hr p.i. in both wild-type CHIKV and MVA-CHIK infected cells, but not in MVA-GFP or mock infected cells. Interestingly, a cleaved E2 protein (∼50 kD) was detected in wild-type infected cells, but not in the MVA-CHIK lysates, suggesting the lack of furin cleavage and release of the E3 peptide from the p62 precursor protein. Expression of E2 was observed in the supernatant of CHIKV infected cells, but there was no detection of E2 expression in MVA-CHIK, suggesting that the protein was not being secreted.

To determine the cellular localization of CHIKV proteins, MVA-CHIK infected cells were fixed and then probed for production of CHIKV E3/E2 proteins using polyclonal serum. In order to determine if the E2 protein was reaching the surface of the cells, a single cell set was permeabilized while the other set remained intact. Permeabilized cells stained much brighter than those that were not (Fig. 1d). This indicated that CHIKV E3/E2 proteins were being maintained inside the cell and not reaching the cell surface as is observed with wild-type CHIKV infection.

### Immunogenicity and protection in A129 mice

**Figure 2:**
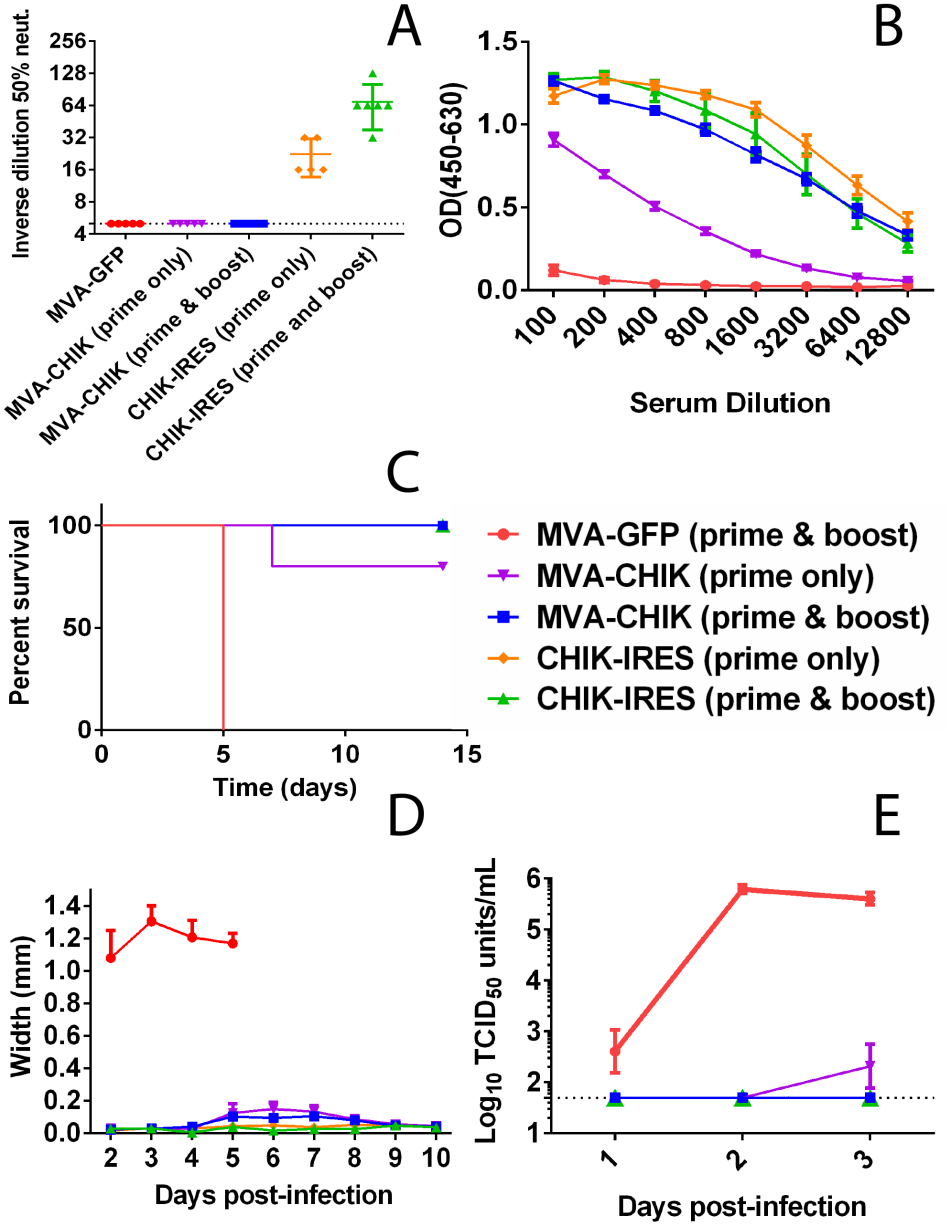
MVA-CHIK candidate vaccine protected A129 mice against CHIK-LR challenge. A129 mice were vaccinated with either prime alone or a prime and boost (day 28) of each vaccine virus (10^7^ TCID_50_ units of MVA-CHIK or MVA-GFP, or 10^4^ TCID_50_ units of CHIK-IRES). Mice were then challenged with 10^2^ TCID_50_ units of wild-type CHIKV intradermally (in the hind left footpad) eleven days post-prime or two weeks post-boost. Prior to boost (where applicable) and challenge, mice were bled and serum was monitored for both total Ig(G+M) and neutralizing activity by TCID_50_ micro-neutralization assay (A) and ELISA (B), respectively. Prime only MVA-GFP data is not shown because it does not differ with the prime & boost groups. Mice were monitored for 14 days following challenge for survival (C) and footpad swelling (D). The first three days following challenge viremia levels were measured via TCID_50_ (E). The dotted line indicates the limit of detection of the assay.

To evaluate the protective efficacy of the MVA-CHIK vaccine, an active immunization study was conducted in A129 mice. This mouse strain is more sensitive to CHIKV infection and experience high viremia, footpad swelling and succumb to infection [51] compared to immunocompetent strains of mice. Mice were vaccinated as described previously and then challenged with wt-CHIKV. MVA-CHIK or MVA-GFP vaccinated mice had background levels of neutralizing antibodies, whereas all CHIK-IRES vaccinated mice seroconverted (Fig. 2a). Serum collected from these mice post-prime and post-boost was tested for total anti-virus Ig(G+M) antibodies by ELISA. A clear booster effect was seen in MVA-CHIK vaccinated mice, as the differences between post-prime and post-boost samples were highly significant (*p*<0.001) (Fig. 2b). This difference was not as profound with CHIK-IRES vaccinated mice as they had high levels of anti-virus antibodies even prior to boost. MVA-CHIK and CHIK-IRES groups were protected 100% from lethal challenge after prime and boost (Fig. 2c). In addition, MVA-CHIK mice were completely protected against both viremia and footpad swelling (Figs. 2d–e).

**Figure 3:**
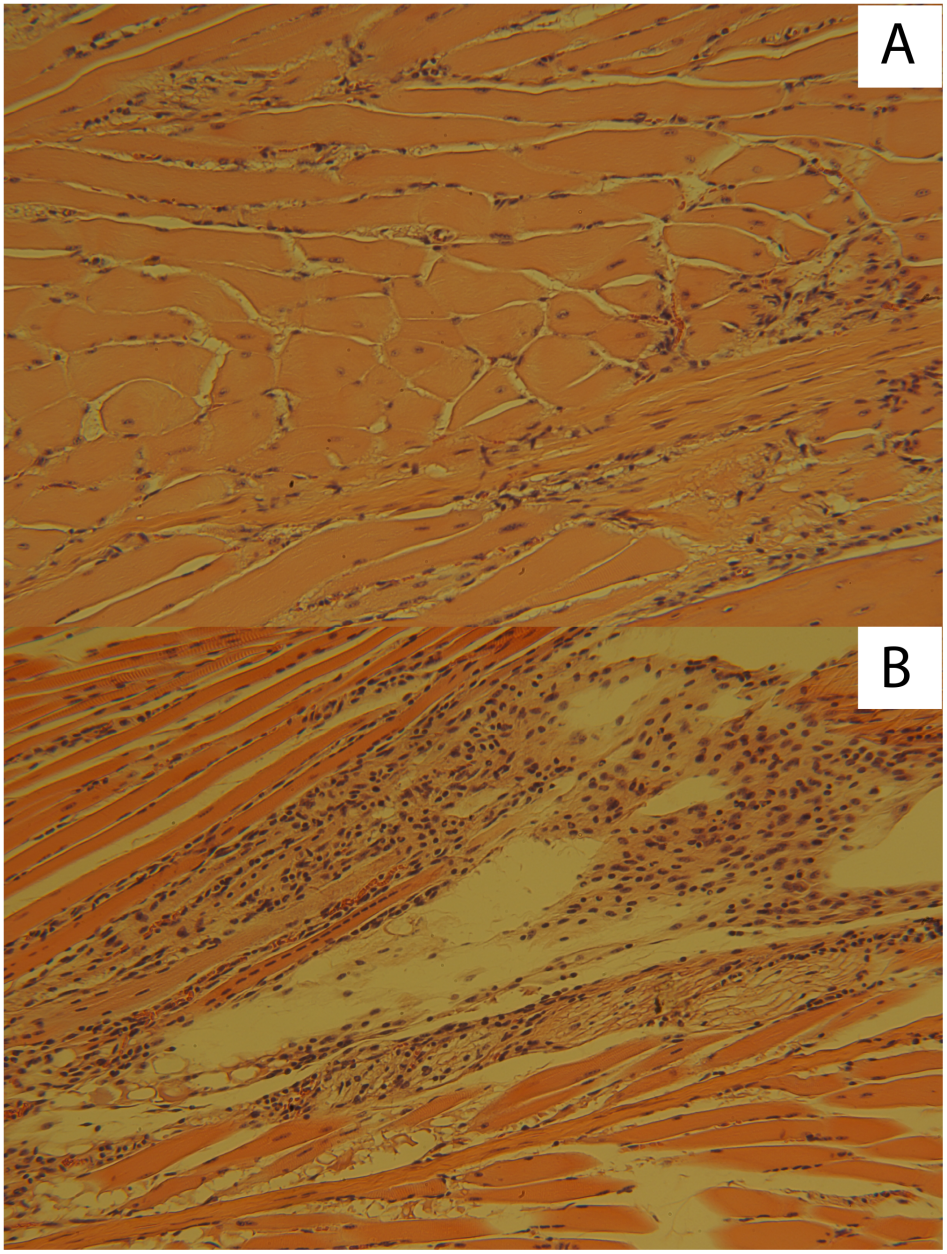
MVA-CHIK vaccine protects against local inflammation following CHIK-LR challenge. 7(H&E). MVA-CHIK vaccinated mice showed low levels of inflammation (A) upon infection with 10^2^ TCID50 units of wild-type CHIKV. MVA-GFP vaccinated mice developed massive inflammation with necrotic muscle degeneration and edema following challenge (B).

Next, we compared by histopathology the footpads of MVA-CHIK and MVA-GFP vaccinated A129 mice. Vaccinated mice were challenged in the same way described above and subsequently were euthanized seven d.p.i. to assess local tissue damage. Following infection, footpads from MVA-CHIK vaccinated mice displayed mild inflammation with limited muscle damage (Fig. 3a). In contrast, footpads from MVA-GFP mice exhibited severe necrotic muscle degeneration with edema, consistent with previous reports from unvaccinated mice (Fig. 3b) [47], [67].

### Immunogenicity and protection in BALB/c mice

**Figure 4:**
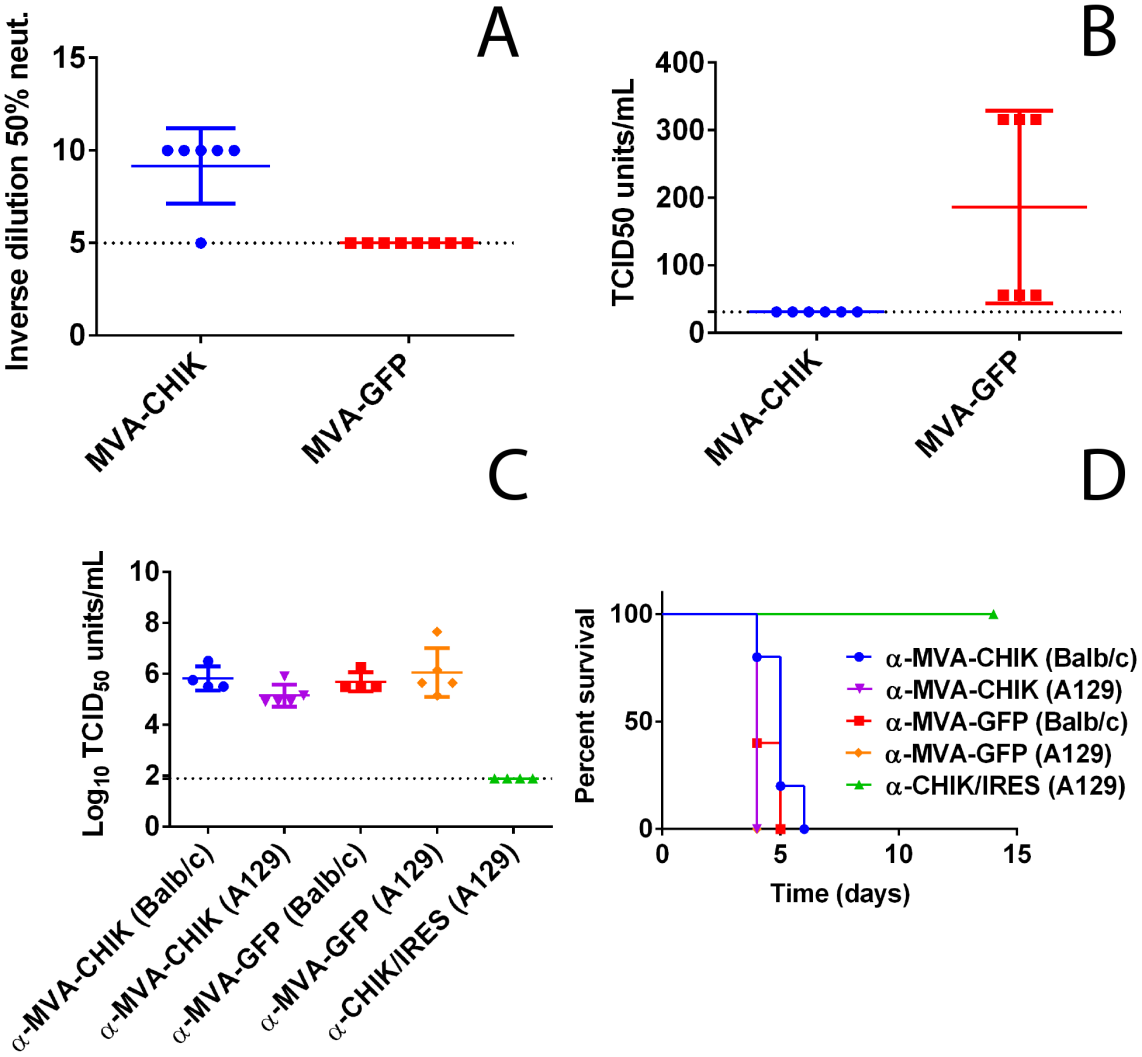
MVA-CHIK candidate vaccine fully protects BALB/c mice against CHIKV viremia. BALB/c mice were vaccinated with a prime and boost (day 28) of 10^7^ TCID_50_ units of MVA-CHIK vaccine virus and then challenged with 10^4^ TCID_50_ units of wild-type CHIKV intradermally (in the hind left footpad) two weeks post-boost. Prior to challenge, BALB/c mice were bled and serum was monitored for neutralizing activity by TCID_50_ micro-neutralization assay (A). Mice were bled two days following challenge and viremia levels were measured via TCID_50_ (B). For passive immunization, prime and boost vaccinated BALB/c and A129 mice were bled and serum taken from each mouse was pooled with equal volumes into separate pools from each strain. Pools of serum (100 µL for BALB/c and 200 µL for A129) were then injected i.p. into A129 mice which were challenged 24 hrs later with 10^2^ TCID_50_ units of wild-type CHIKV. Viremia was measured 2 d.p.i (C) and survival was monitored for two weeks post-challenge (D). The dotted line indicates the limit of detection of the assay.

The immunocompetent BALB/c mouse model was selected to evaluate the immunogenicity of MVA-CHIK as it has been used previously with MVA and CHIKV [37], [53], [68]. Following vaccination, low levels of neutralizing antibodies were detected in mice immunized with MVA-CHIK (Fig. 4a). Immunized mice were then challenged with wild-type CHIKV to determine protection against viremia. Viremia was not detected in any of the MVA-CHIK vaccinated mice (n = 6) (Fig. 4b). In contrast, all MVA-GFP immunized mice had significant levels of viremia (*p* = 0.027).

### Passive transfer of immune serum

To determine whether antibodies present in BALB/c (Avg. neut. titer of 9.2) or A129 (undetectable neut. titer) vaccinated mice were sufficient for protection against CHIKV, pooled immune serum was passively transferred into naïve A129 mice. As a positive control, an additional group of mice was treated in the same manner with a pool of serum (avg. neut. titer of 64) from A129 mice vaccinated with CHIK-IRES candidate vaccine. All of the mice treated with either anti-MVA-CHIK or MVA-GFP immune serum had high levels of viremia and succumbed to infection (Fig. 4c–d). Viremia in mice passively transferred serum from MVA-CHIK vaccinated A129 was slightly reduced on day 2 post-infection, however this was not significant (p = 0.11). In contrast, CHIK-IRES immune serum provided full protection against viremia and mortality (Figs. 4c–d).

### MVA-CHIK immune CD4^+^ T cells are indispensable for protection in the A129 model

**Figure 5:**
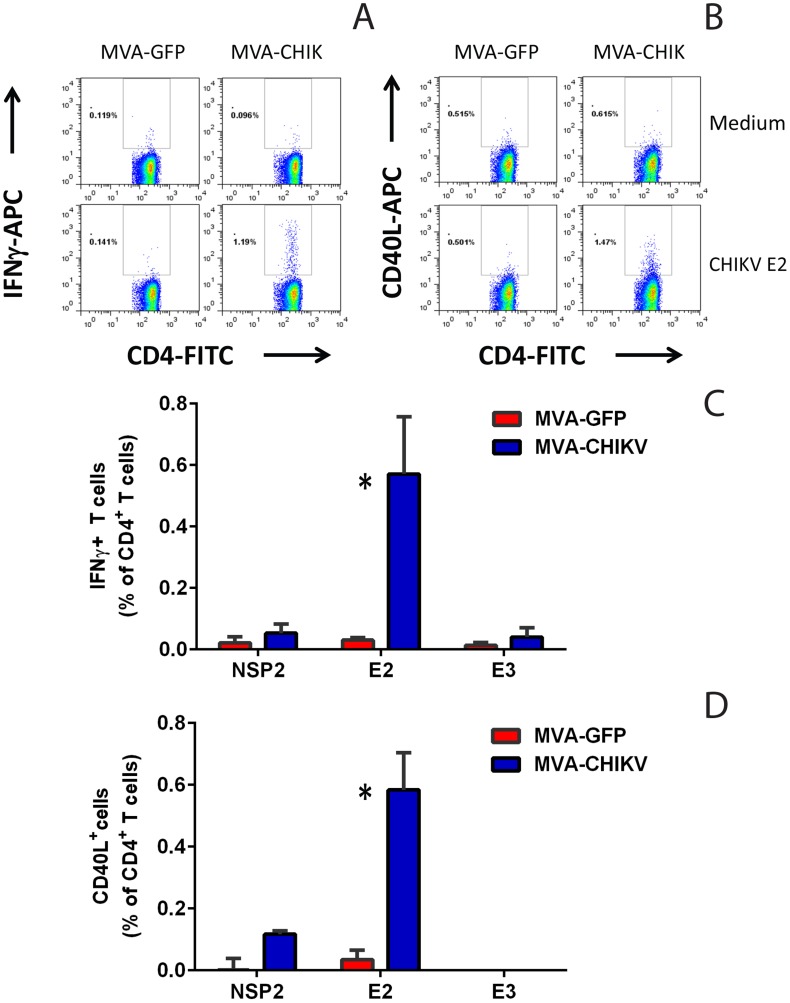
MVA-CHIK elicited a CHIK-E2-specific CD4^+^ T cell response in A129 mice. Two weeks following boost, splenocytes from MVA-GFP or MVA-CHIK immunized mice were stimulated with CHIKV NSP2, E2 or E3 peptide pools (1 µg/well). T-cells were then stained for intracellular IFNγ (A and C) or CD40L (B and D). Representative dot plots (for only one mouse per group) are shown for both cytokines with MVA-CHIK and MVA-GFP stimulated with E2 peptide pools or background media control (A and B). For stimulation with E2, E3 and NSP2 peptide pools data are presented as the mean+SD of the percentages cytokine-positive cells among gated CD4^+^ T cells (3 mice/group) with background subtracted (C and D). Stimulation was significantly higher for IFNγ and CD40L in the MVA-CHIK immune group, as compared to MVA-GFP control (p = 0.02 and p = 0.01, respectively). No cytokine production in CD8^+^ T cells was observed (data not shown).

To determine the role of T cells in protection we first investigated whether MVA-CHIK could elicit a T cell response in A129 mice by measuring cytokine production. Upon *in vitro* re-stimulation with a CHIKV peptide pool that covers the protein sequence of E2, MVA-CHIK immune CD4^+^ T cells produced the cytokine IFNγ and upregulated co-stimulatory molecule CD40L at significantly higher levels as compared to MVA-GFP vector control (p = 0.02 and 0.01, respectively) (Fig. 5a–b). No response was observed when MVA-CHIK splenocytes were stimulated with peptide pools against either E3 or NSP2 (viral protein negative control) (Fig. 5c–d), suggesting the immune response is specific to E2. In addition, there were no significant differences in other cytokines monitored, including TNFα, IL-2, IL-4, IL-6, IL-10, and Il-17a (data not shown). Furthermore, none of the cytokines tested showed a difference between the two groups in immune CD8+ T cells (data not shown). Similar results were obtained when MVA-CHIK immune splenocytes were stimulated with live or inactivated whole virus preparations (data not shown).

**Figure 6:**
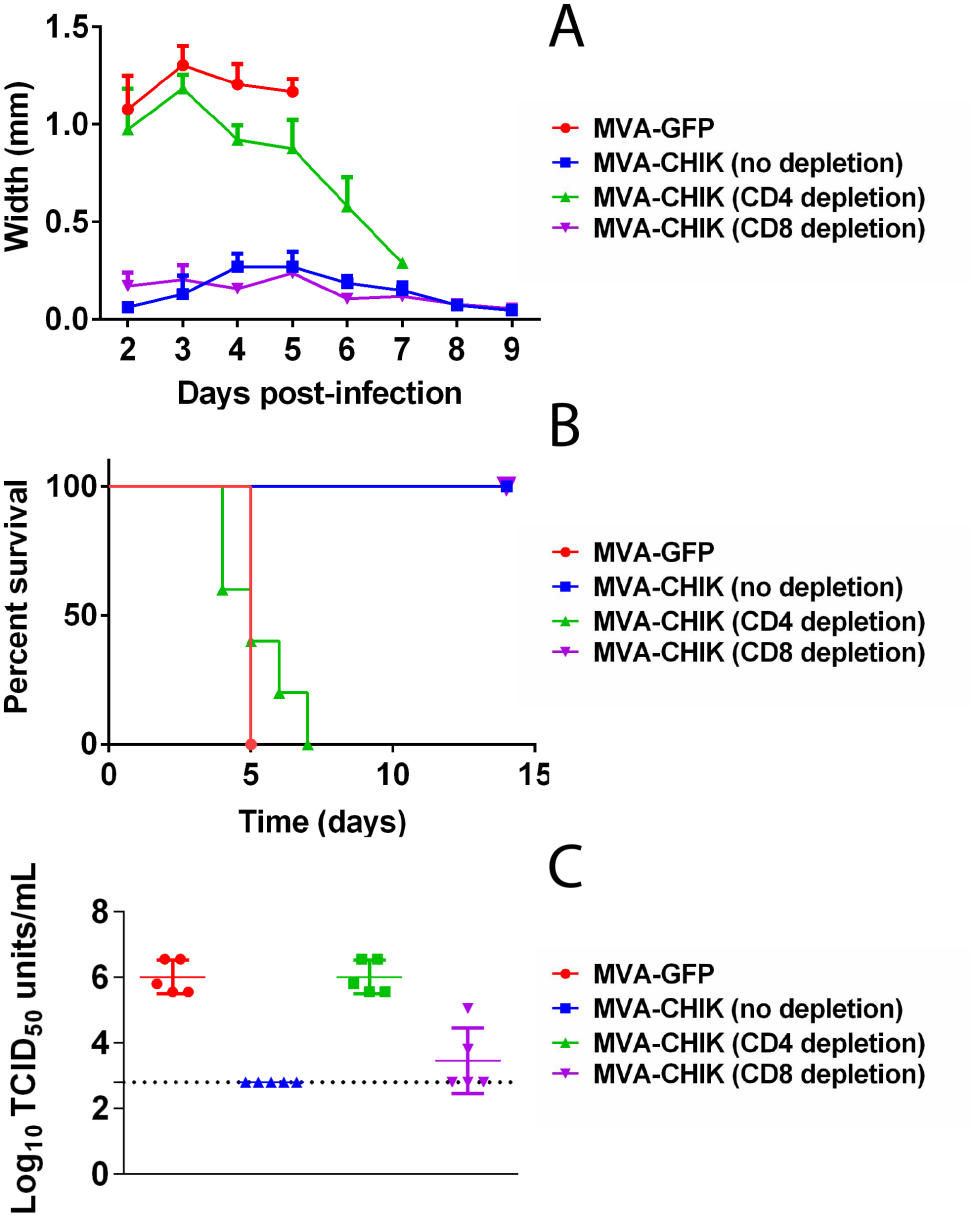
Depletion of MVA-CHIK immune CD4^+^ T cells, but not CD8^+^ T cells, abolished the protective immunity afforded by MVA-CHIK in A129 mice. A129 mice were depleted of CD4^+^ or CD8^+^ T cells with anti-CD4 or CD8 mAbs and were then challenged with 500 TCID50 units of CHIK-LR i.d. via the left hind footpad on day 14 post boost. Mice were monitored for (A) footpad swelling; (B) survival and (C) Viremia (measured 2 d.p.i by TCID50 assay). The dotted line indicates the limit of detection of the assay.

To test whether MVA-CHIK-specific immune CD4^+^ or CD8^+^ T cells were indeed protective, we depleted CD4^+^ or CD8^+^ T cells from vaccinated mice prior to challenge with wt-CHIKV. Mice depleted of CD8^+^ T cells or control non-depleted mice had no footpad swelling and survived the challenge (Fig. 6a–b). In contrast, CD4^+^ depleted mice along with MVA-GFP controls succumbed to infection (Fig. 6b). Mice depleted of CD4^+^ T cells also had high levels of viremia and footpad swelling, similar to the MVA-GFP controls (Fig. 6a–c). Interestingly, CD8^+^ T cell depleted vaccinated mice remained healthy without footpad swelling throughout the duration of the study despite developing a low level of viremia three d.p.i.

## Discussion

The recent re-emergence of CHIKV has resulted in several explosive outbreaks and underscores the need for an effective vaccine. Several groups have recently employed varying strategies to construct effective vaccines against CHIKV. Live-attenuated, inactivated, adenovirus vectored, DNA, and VLP based strategies all have been tested and have been shown to be effective in providing protection against CHIKV [12], [13], [68], [69]. Despite promising results, these vaccine candidates all have some drawbacks. Safety, stability, and production of a broad long-lived immune response are necessary for any effective vaccine. Safety concerns remain for both live-attenuated and adenovirus based approaches. Inactivated and VLP based approaches require adjuvants and normally elicit weak T cell responses. In contrast, MVA has been shown to be safe in thousands of human patients and also in every animal tested [21], [22], [58]. Furthermore, mice deficient in α/β/γ interferon signaling experienced no clinical symptoms following vaccination with MVA-CHIK (data not shown) and we have previously shown other MVA constructs to be safe in highly immunodeficient SCID mice [37]. In addition, the stability of poxviruses and MVA's ability to be lyophilized are optimal for use in developing countries where the vaccine is needed most and cold-chains cannot be reliably maintained. MVA also has been shown to stimulate a broad immune response, including the induction of cellular immunity [62], [70].

Here, we describe the construction, expression, and preclinical efficacy and safety of a novel MVA-CHIK vaccine candidate. BALB/c mice, which are immunocompetent, were 100% protected from viremia upon challenge. A129 mice, which are deficient in α/β interferon signaling, and therefore highly susceptible to CHIKV infection, were also completely protected against challenge with wtCHIKV after prime and boost vaccinations. In addition, 80% of mice were protected from lethal challenge following prime only vaccination with MVA-CHIK. Despite the robust protection observed, mice did not produce a strong neutralizing antibody response following vaccination. Although vaccinated A129 mice developed undetectable levels of neutralizing antibodies, they did produce a significant amount of anti-virus antibodies, which for other alphaviruses have been shown to be protective [71]–[73]. A129 mice passively transferred MVA-CHIK immune serum from either BALB/c or A129 mice were not protected against mortality, or footpad swelling following challenge, despite a slight reduction in viremia 2 days post-infection in mice transferred A129 MVA-CHIK immune serum (p = 0.11). This suggests antibodies induced by vaccination were not sufficient to provide protection in this manner. This could be due to the fact that passively administered immune serum is significantly diluted in the circulation of naïve mice. Further evidence that antibodies are not sufficient for protection alone was provided by depletion studies conducted in MVA-CHIK vaccinated mice. When immune CD4^+^ T-cells were depleted, despite the presence of full levels of circulating anti-virus antibodies, mice were 100% susceptible to lethal infection. If anti-virus antibodies were essential for protection a reduction in mortality or viremia would be expected in the CD4+ depleted mice, which was not observed. These data suggest that MVA-CHIK is providing protection by a novel mechanism that is not based solely on vaccine induced serum circulating antibodies.

While antibodies do not protect via passive transfer or during depletion, it remains unclear what role they may be playing during infection of an MVA-CHIK immunized mouse. Immunocytochemistry and western blot analysis suggested CHIKV p62 protein was being maintained inside of the cell and that furin cleavage was not occurring to separate E3 from E2, respectively. P62 was chosen due the presence of immunodominant epitopes and the fact that previous reports have shown that it could be an effective immunogen against other alphaviruses [71], [74]. In addition, it has been previously observed that when expressed by a plasmid, the presence of E1 is not a requirement to allow E2 to be placed on the outside of the plasma membrane [75], [76]. Barth et al. (1997) also demonstrated that in the context of an E1 deleted Semliki Forest virus, p62 is not transported efficiently to the plasma membrane while E2 alone is [77]. Therefore, absence of E2 on the surface of infected cells is not completely unexpected and may have proven to be serendipitous in allowing the production of a more robust CD4^+^ immune response. This could provide an explanation for the lack of a strong humoral immune response, as antibodies would have limited access to this intracellular protein. In addition, an improperly folded protein would be expected to produce many virus binding antibodies but few that are neutralizing, which our data suggest. A recent report by Metz et al. (2013), suggested that E2 produced in combination with E1 in a baculovirus system is more immunogenic (i.e., produces higher neutralizing antibodies) than either protein alone [78]. This might suggest that E1 and E2 are more efficient together to obtain a robust neutralizing antibody response and might explain why MVA-CHIK produces few neutralizing antibodies.

Analysis of *ex vivo* cellular responses indicated that MVA-CHIK induced antigen-specific CD4^+^ T cells but not CD8^+^ T cells, suggesting that immune CD4^+^ T cells are the main effector cells. This is supported by the cellular depletion studies that showed the protective role of CD4+ T-cells. T-cell mediated protection has previously been demonstrated with another alphavirus, VEEV [79]. Yun et al. (2009) were able to adoptively transfer CD4^+^ T-cells and obtain protection after multiple immunizations, where passive transfer of hyper-immune serum was not protective. Interestingly, they did not observe a difference in viral titers in the brain from surviving mice, while MVA-CHIK vaccination completely protected against viremia in our studies.

Recent studies based on another vaccine candidate, CHIK-IRES, have shown that a correlate of protection based on antibodies can be established for CHIKV [50]. Herein, we present data that suggest that this is specific to the vaccine being used and that there may be more than one way to provide protection against CHIKV. MVA-CHIK selectively expresses E2-E3 proteins. It could be argued that the processing of this virus is substantially different than the live attenuated CHIK-IRES vaccine or wild-type CHIKV. As a result, during infection a different set of epitopes are generated that may well be protective via alternative mechanisms. In another report, using mice deficient in either B-cells or CD4^+^ T cells, data were presented that suggested that antibodies are essential in controlling CHIKV infection and that CD4^+^ T cells may exacerbate disease [49], [80]. Hawman et al. (2013) found similarly, that mice deficient in both B and T-cells had less severe tissue pathology early after CHIKV infection when compared to wild-type controls [81]. However, in the same report it was demonstrated that virus was inefficiently cleared in the absence of lymphocytes, suggesting a possible dual role for these cells during early CHIKV infection where some tissue damage is allowed in order to control viral dissemination. While we observe very little evidence of tissue damage in vaccinated mice, it is possible that MVA-CHIK induced specific CD4^+^ T-cells which are able to effectively control viral replication while minimizing collateral tissue injury. It remains possible that CD4 T-cells are required for recall responses to produce antibodies following challenge, although this is unlikely as mice would be expected to have early viremia, which we did not observe in vaccinated mice (Fig. 2E). Currently, we are conducting studies to determine the role of CD4^+^ T cells in protection. Our data are also contrary to the previous report by Teo et al. (2013), where no footpad swelling was observed upon antibody depletion of CD4^+^ T cells during CHIKV infection [80]. However, their studies were performed in naïve mice with a functional interferon system, in contrast, our studies used vaccinated mice without proper IFN α/β signaling, and thus might account for the differences observed. Additionally, recent studies in humans show that there is a strong CD4+ IFNγ positive T-cell response directed against the E2 protein, suggesting it might be playing a role in the protective immune response against the virus [82]. Therefore, the data presented here may be relevant to human infection, and suggest that antibodies may not be the only mediator of protection against alphaviruses.

A recently published report by Garcia-Arriaza et al. (2014) also has used MVA as a vector as a CHIKV vaccine candidate [83]. Their vaccine, which expressed the entire structural protein is effective at inducing a high level neutralizing antibody response accompanied by a strong CD8+ T-cell specific cell mediated response, in direct contrast to the results reported here. They report that the E2 protein can be found in both the plasma membrane and the cytoplasm, in contrast to our results which clearly showed E2 is not on the surface of the cell (Figure 1). This could explain why the immune responses are so drastically different between the two vaccine candidates. While depletion studies indicated CD4+ T cells are indispensable for protection for MVA-CHIK in this report, the mechanism of protection is unclear in their report, although high levels of neutralizing antibodies induced by their vaccine are likely protective [50]. Additionally, the vaccine constructed by Garcia-Arriaza et al. (2014) uses the entire structural protein consisting of Capsid-E3-E2-6K-E1. In contrast, our vaccine contains only the E3-E2 portion. Recent reports have shown that the capsid protein contains immunodominant epitopes for both B and T cells [55], [82], and was therefore excluded from our vaccine construct in favor of E2 with its accessory protein E3. Furthermore, we used a footpad injection which more closely mimics intradermal vaccination a human might be expected to receive. Garcia-Arriaza et al. (2014) used intraperitoneal vaccinations, which are not given to humans and therefore could potentially induce a vastly different immune response delivered by another route.

In conclusion, these studies demonstrate the potential of MVA to effectively express CHIKV E3-E2 proteins and generate protective immune responses despite the presence of low or absent neutralizing antibody responses. The MVA construct expressing CHIKV p62 protein was effective in several mouse models and provided protection against two critical markers of disease, viremia and joint swelling. The vaccine was protective despite low levels of neutralizing antibodies which are normally considered to be the golden standard for protection against alphaviruses. Therefore the results reported here challenge the assumption that only these antibodies are effective in providing protection against CHIKV or other alphaviruses. Future studies combining a T-cell targeted vaccine, like the one presented here, along with a more traditional vaccine which induces strong neutralizing antibodies could provide a more robust and comprehensive immune response in a safe and easy to deliver vaccine regimen. However, further work is required to characterize the protective mechanisms it provides.
